# Flowering pathway genes: key targets for accelerated breeding in woody plants

**DOI:** 10.1007/s00299-025-03690-9

**Published:** 2025-12-28

**Authors:** Guo-qing Song, Jirapa Jaikham, Weiqi Wang

**Affiliations:** Plant Biotechnology Resource and Outreach Center, Department of Horticulture, Michigan State University, East Lansing, MI 48824 USA

**Keywords:** Breeding, Florigen, Floral initiation, Flowering mechanism, Gene-editing-hatcher, Juvenility

## Abstract

Conventional breeding of many woody plants through hybridization is time-consuming in comparison to annual plants. This delay is primarily attributed to their lengthy juvenile phase, which typically spans multiple years depending on the specific crop before they are capable of blooming. Over the past two decades, significant efforts have been dedicated to deciphering the molecular mechanism of flowering and to accelerating woody plant breeding, also known as FasTrack breeding, by shortening juvenility. This has been achieved through the utilization of cutting-edge technologies such as genetic engineering of key flowering-pathway genes. By consolidating previous research and outlining potential candidate genes, this review discusses relevant strategies for FasTrack breeding to provide a foundational insight into accelerating woody species improvement via gene editing.

## Introduction

Climate change poses a significant challenge to the global agriculture. The increasing climate instability, driven mainly by rising temperatures and increased CO_2_ levels, not only leads to an increase in extreme weather events but also influences the prevalence and distribution of pests and diseases, and ultimately brings increased uncertainty for the productivity of global agriculture (Razzaq, et al. 2021). Among the possible solutions to address the challenges posed by climate change, including the development of climate-resilient crop varieties, the implementation of sustainable farming practices, and the enhancement of water management strategies, the foremost is the development of climate-resilient crop varieties. Therefore, the application of genetic enhancement in agronomic traits related to both crop yield and quality through plant breeding emerges as a crucial solution to address the diverse challenges arising from climate change.

**Fig. 1:**
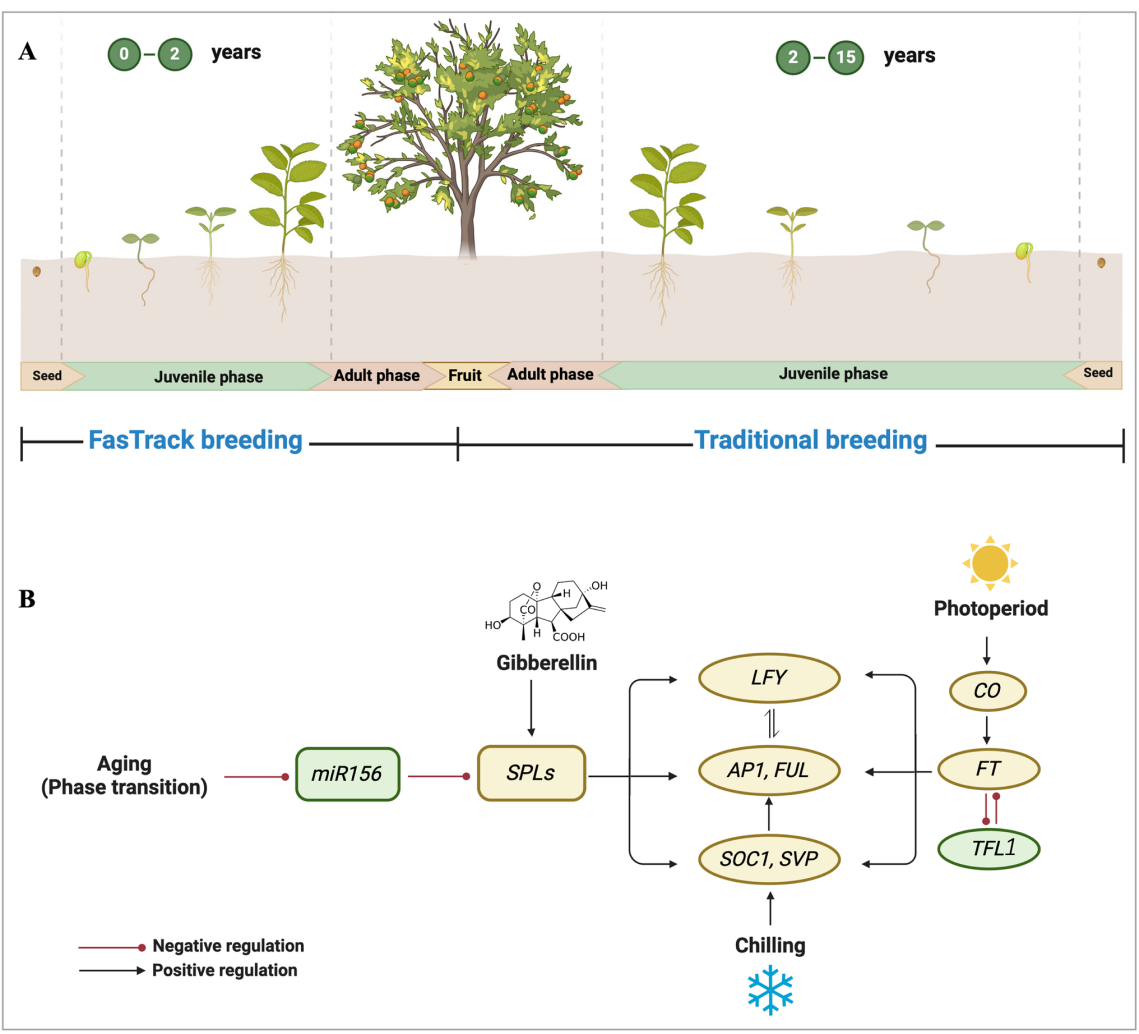
FasTrack breeding to shorten the juvenile phase in woody plants. **A** Schematic of FasTrack breeding coupled with genetic engineering. **B** Potential flowering-pathway genes for application in FasTrack breeding. Panel A was created with BioRender

Woody plants breeding through hybridization, including fruit and forest trees, is of importance in modern agricultural systems for new varieties development. Although aims and process differ between fruit and forest trees, the prolonged juvenility period is always the key barrier to the release of new cultivars in woody perennial plants breeding projects. Juvenility period refers to the extended phase of vegetative growth that occurs before the plant reaches reproductive maturity (adult phase) (Fig. 1). This juvenile phase usually lasts for several years or even decades and can be influenced by various factors, including environmental conditions, genetics, and cultivation practices (Hackett 2011). For example, the juvenile phase for many *Prunus* fruits trees (such as peach, plum, apricot, and cherry) is often beyond 3 years (Dirlewanger et al. 2007; Grossman and DeJong 1994; Neumüller 2011); citrus plants, such as orange, lemon, mandarin, and tangerine, have a juvenile period ranging from 5 to 10 years (Agustí, et al. 2022); and the juvenile phase of apple and pear trees usually persists for 6–12 years (Visser 1965; Zimmerman 1975). Generally, to develop and release a new cultivar typically demand an average of 10–30 years, with the breeding process remaining slow and costly (Flachowsky, et al. 2009). Obviously, shortening juvenile phase is desirable to expediting breeding efforts (Pan, et al. 2023).

Advancements in genetics, genomics, and molecular techniques have driven significant progress for accelerating woody plant breeding process, primarily by shortening lengthy juvenile phase, an approach often termed “FasTrack breeding”. It is important to distinguish this from “Speed breeding”, a term more commonly used to annual plants. Also known as the Rapid Generation Advance, speed breeding was first conceptualized for crop improvement in the 1930s (Wanga, et al. 2021; Watson, et al. 2018). Essentially, it employs tools like marker-assisted selection, genomic selection, and controlled environments to reduce generation times. However, several speed breeding techniques, such as those involving the regulation of environmental factors to shorten breeding cycles (Abdul Fiyaz et al. 2020; Wanga, et al. 2021) and double haploid techniques (Andersen 2005; Das, et al. 2018; Germana 2011), are often unsuitable for perennial woody plants. In this review, FasTrack breeding specifically refers to the use of genetic engineering to directly manipulate flowering-pathway genes, thereby shortening juvenility and accelerating the breeding cycle (Fig. 1A).

To date, several reviews have documented the application of FasTrack breeding for woody plants (Callahan, et al. 2016; van Nocker and Gardiner 2014). Built upon this information, as well as our recent and extensive research in this area, we have compiled this review to update previous findings, reveal potential candidate genes, and discuss relevant strategies for FasTrack breeding (Fig. 1B). Ultimately, this review offers insights into accelerating breeding efforts, particularly in woody species (Table 1).

**Table 1:** **A** Literature survey on the functional analysis of major flowering-pathway genes that promote flowering

Flowering gene	Gene expression system	Genetic manipulation (gene source)	Transgenic plant	Phenotype of transgenic plant	Reference
Aging pathway *miR156*	Constitutive expression (CX)	pCaMV 35S (p35S)-*miR156* (Poplar)	Poplar	o Prolong the juvenile phase o Delay the growth cession	(Liao, et al. 2023; Wang, et al. 2011)
		p35S-*MIR156* (Citrus)	*Arabidopsis*	o Repress flowering	(Shalom, et al. 2015)
Aging pathway *SPLs*	CX	p35S-*CiSPL5* (Citrus)	*Arabidopsis*	o Promote flowering	(Shalom, et al. 2015)
		p35S-*JcSPL3* (*Jatropha*)	*Arabidopsis*	o Early flowering o Alter the morphology	(Yu, et al. 2020)
		p35S-*EjSPL3*, p35S-*EjSPL4*, p35S-*EjSPL5*, p35S-*EjSPL9* (Loquat)	*Arabidopsis*	o Promote flowering	(Jiang, et al. 2019)
		p35S-*FhSPL5*, p35S-FhSPL9, p35S-FhSPL11 (Citrus)	*Arabidopsis*	o Early flowering	(Li, et al. 2023)
PEBP genes *FT*	CX	p35S-*MdFT1* (Apple)	Poplar clone W52	o Early flowering o Morphological changes in inflorescences	(Tränkner, et al. 2010)
		p35S-*MdFT1*	Apple ‘Pinova’ seedling clone	o Early flowering o Morphological changes	(Tränkner, et al. 2010)
		p*rolC*-*AtFT* (Arabidopsis)	Apple rootstock ‘JM2’	o Early flowering o Morphological changes in inflorescences and architectures	(Tanaka, et al. 2014)
		p35S-*AtFT*	Apple rootstock ‘JM2’	o In vitro flowering o Fail to obtain transgenic plants	(Tanaka, et al. 2014)
		p35S-*MdFT1*	Apple rootstock ‘JM2’	o Early flowering o Morphological changes in inflorescences and architectures o Reduced vigorous growth	(Kotoda, et al. 2010)
		p35S-*PcFT2* (Pear)	Apple ‘Galaxy’	o Not early flowering o Less sensitive to short-day-induced dormancy	(Freiman, et al. 2015)
		p35S-*PcFT2*	Tobacco	o Early flowering	(Freiman, et al. 2015)
		p35S-VcFT (Blueberry)	Blueberry ‘Aurora’	o Early flowering o Morphological changes	(Song 2025; Song, et al. 2013)
		p35S-*CiFT* (*Citrus unshiu* Marc.)	Trifoliate orange	o Early flowering and fruiting o Reduce generation time o Morphological changes	(Endo, et al. 2020, 2005)
		p35S-*AtFT* p409S *Ubiquitin*-*AtFT*	Eucalyptus hybrid SP7	o Early flowering o Morphological changes in architectures o Highly branched o Viable pollen grains	(Klocko, et al. 2016a, b)
		p35S-*AcFT1*, p35S-*AcFT2* (Kiwifruit)	Kiwifruit	o In vitro flowering o Fail to obtain transgenic plants	(Voogd, et al. 2017)
		p35S-*CiFT*	European pear	o Early flowering	(Matsuda, et al. 2009)
		p35S-*PtFT1* (Poplar)	Plum	o Early flowering o Continuous flowering o Not enter dormancy after cold or short-day treatments o Morphological changes in inflorescences and architectures	(Callahan, et al. 2016; Srinivasan, et al. 2012)
		p35S-*PsFT1*, p35S-*PsFT2* (*Populus simonii*)	Poplar clone T89	o Early flowering	(Böhlenius, et al. 2006; Shen, et al. 2012)
		p35S-*PtFT2* (Poplar)	Poplar clone 717-1B4	o Early flowering	(Hsu, et al. 2006)
	Tissue-specific expression	p*AtSUC2*-*CcFT3* (*Citrus clementina*)	Carrizo citrange rootstocks	o Early flowering o Normal morphology and vigor	(Soares, et al. 2020)
		p*AtSUC2*-*MdFT1*	Apple ‘Pinova’ seedling clone	o Not produce flowers during the first season o Normal vigor	(Tränkner, et al. 2010)
	Heat shock-inducible expression	p*HSP*-*FT1*, p*HSP*-*FT2* (*Populus*) p*HSP*-*AtFT*	Aspen hybrid clones INRA 353–53 and INRA 717-1B4	o Daily heat shock could induce flowering	(Zhang, et al. 2010)
		p*HSP*-*AtFT*	Aspen hybrid clones INRA 353–53 and INRA 717-1B4	o Repeated heat induction could accelerate flowering	(Azeez and Busov 2019)
		*HSP-AtFT*	Aspen clone W52 and aspen hybrid clone Esch5	o Chilling is required to induce fertile flowers	(Hoenicka, et al. 2016, 2014)
		p*HSP*-*PtFT1,* p*HSP*-*PtFT2* (Poplar)	Apple ‘Pinova’	o Daily heat shock could induce flowering o Normal morphology of flowers o Fertile flowers	(Wenzel, et al. 2013)
		p*HSP*-*PtFT1*	*Eucalyptus* hybrid SP7	o Early flowering o Normal vigor	(Klocko, et al. 2016a, b)
	Virus-mediated expression	ALSV-*AtFT* ALSV-*MdFT1*	Apple seedlings	o Precocious flowering o Normal inflorescences o Viable pollen grains	(Yamagishi, et al. 2011)
		ALSV-*AtFT* ALSV-*MdTFL1* (Apple)	Apple seedlings	o Higher efficiency of early flowering induction	(Yamagishi, et al. 2014)
		ALSV-*AtFT* ALSV-*PcTFL1* (Pear)	Pear seedlings	o Early flowering induction o Continuous flowering o Fruiting upon pollination	(Yamagishi, et al. 2016)
		clbvINpr-*AtFT* clbvINpr-*CiFT*	*Citrus*	o Early flowering induction o Normal plant architecture o Normal inflorescences o Fruiting ability	(Velázquez, et al. 2016)
PEBP genes *TFL1*	CRISPR/Cas9 gene editing	*AcCEN4* and *AcCEN* (Kiwifruit)	Kiwifruit	o Rapid terminal flower and fruit setting o Compact plant with	(Varkonyi-Gasic, et al. 2019)
		*MdTFL1.1* (Apple)	Apple	o Early flowering	(Charrier, et al. 2019)
		*PcTFL1-1* and *PcTFL1-2* (Pear)	Pear	o Early flowering o Reducing vegetative growth vigor	(Freiman, et al. 2012)
	RNAi	*MdTFL1-1*	Apple seedling	o Early flowering o Reduce gemination time	(Flachowsky, et al. 2012)
		*PopCEN1* and *PopCEN2*	*Populus*	o Indeterminate roles in terminal vegetative meristems	(Mohamed, et al. 2010)
		p35S-*LFY* (Arabidopsis)	o Arabidopsis o Aspen (*Populus tremula* × *tremuloides*)	o Precocious flowering	(Weigel and Nilsson 1995)
MADS-box genes *AP1*	CX	p35S-*BpAP1* (Birch)	Birch	o Early flowering o Dwarfism	(Huang, et al. 2014)
		p35S-*AP1* (*Salix integra* Linn.)	Poplar	o Early flowering	(Yang, et al. 2017)
		p35S-*JcAP1* (*Jatropha*)	*Jatropha*	o Little variation in the flowering time	(Tang, et al. 2016)
		p*AtRD29A*-*CsAP1* (Sweet orange)	*Citrus*	o Early flowering	(Orbovic, et al. 2021)
	Stress-inducible expression	p35S-*BpMADS4* (Birch)	Birch	o Accelerates flowering	(Elo, et al. 2007)
MADS-box genes *FUL*	CX	p35S-*BpMADS4*	Apple	o Early flowering o Normal morphology	(Flachowsky, et al. 2007a, b; Flachowsky, et al. 2011)
		p35S-*BpMADS4*	Populus	o Changes in senescence and winter dormancy o No early flowering	(Hoenicka, et al. 2008)
		p35S-*BpMADS4*	Pear	o Early flowering o Normal morphology	(Tomes, et al. 2023)
		p35S-*SaFUL* (White mustard)	The hybrid poplar clones 717-1B4	o No early flowering o Significantly restricted plant growth	(Bruegmann and Fladung 2019)
		p35S-*MADS12* (poplar)	The hybrid poplar clone 717-1B4	o No early flowering o Early bud break in ecodormant poplars	(Gómez-Soto, et al. 2021)
MADS-box genes *SOC1*	CX	p35S-*AtSOC1* (Arabidopsis)	The hybrid poplar clones 717-1B4	o No early flowering o Significantly restricted plant growth	(Bruegmann and Fladung 2019)
		p35S-*AcSOC1a* ~ *i*	Kiwifruit	o *AcSOC1e*, *AcSOC1i*, and *AcSOC1f* failed to induce precocious flowering in *Actinidia chinensis* o *AcSOC1i* reduced the duration of dormancy	(Voogd, et al. 2015)
		p35S-*AcSOC1a* ~ *i*	Arabidopsis	o All are able to rescue the late flowering phenotype of the soc1-2 mutant	(Voogd, et al. 2015)
		P35S*-PbSOC1d* and *P35S-PbSOC1g* (Pear)	Arabidopsis	o Early flowering	(Liu, et al. 2020)
		p35S-*VcSOC1*	Tobacco, Blueberry	o Increase the numbers of flower buds in chilled transgenic blueberry o Increase yield potential o Promote flowering in transgenic tobacco	(Song and Chen 2018)
MADS-box genes *SVP*	CX	Apple *SVP* (*SHORT VEGETATIVE PHASE*)	Apple	o Precocious flowering	(Wu, et al. 2021)
		p35S-*AcSVP2*	Kiwifruit	o Prevent premature bud break	(Wu, et al. 2017)
Photoperiod pathway *CO*	CX	p35S-*CO1*/*CO2* (Cottonwood poplar)	Poplar clone 717-1B4	o No significant change in flowering time o Reduced plant size	(Hsu, et al. 2012)
*FD*	CX	p35S-*PtFD* (Silver poplar)	Poplar clone 717-1B4	o Precocious flowering o Morphological changes with a bushy architecture	(Parmentier-Line and Coleman 2016)
*LFY*	CX	p35S-*LFY* p35S-*PtLFY* (*Populus trichocarpa*)	o The hybrid poplar clone 717-1B4 (*P. tremula* × *P. alba*) o *P. tremula* × *P. tremuloides* hybrid clone o Arabidopsis	o *PtLFY* cDNA in *Arabidopsis* accelerated flowering, only one of the many tested transgenic lines of *Populus* flowered precociously	(Rottmann, et al. 2000)
		p35S-*LFY*	Apple	o No early flowering o Columnar phenotype with shorter internodes	(Flachowsky, et al. 2010)
		p35S-*LFY*	*Citrus*	o Early flowering o Normal phenotype	(Peña, et al. 2001)
		p*AtRD29A*-*CsLFY* (*Citrus sinensis*)	*Citrus*	o Early flowering	(Orbovic, et al. 2021)
	Stress-inducible expression	p35S-*AP1* (Arabidopsis)	*Citrus*	o Early flowering o Normal phenotype	(Peña, et al. 2001)

Shaded cells indicate instances where FasTrack breeding has been successfully applied

## Potential genes to shorten juvenile phase

The key for FasTrack breeding in woody plants is to shorten the juvenile phase and thus promote conversion to the adult phase (Fig. 1A). As revealed in proposed regulatory network of flowering, the candidate genes for this transition are mainly in the aging pathway, where *microRNA156* (*miR156*), *microRNA172* (*miR172*), and *SQUAMOSA PROMOTER BINDING PROTEIN-LIKE* (*SPLs*) genes are the primary regulators (Song, et al. 2024; Teotia and Tang 2015). The expression of *miR156*, which is downregulated as the plant ages, facilitates the maintenance of the juvenile phase by suppressing the expression of *SPLs* (Hyun, et al. 2017; Teotia and Tang 2015; Wang, et al. 2009; Wu, et al. 2009; Xu, et al. 2016). This *SPL* family genes often promote floral transition by upregulating the expression of flowering-promoting genes as floral meristem identity genes *LEAFY* (*LFY*) and *APETALA1* (*AP1*) and *SUPPRESSOR OF OVEREXPRESSION OF CONSTAN 1* (*SOC1*) (Albani and Coupland 2010; Lee and Lee 2010; Ma, et al. 2021). In addition to the specific aging pathway and its associated genes, Phosphatidyl Ethanolamine-Binding Proteins (*PEBPs*), such as *FLOWERING LOCUS T* (*FT*) and *TERMINAL FLOWER 1* (*TFL1*), have also emerged as noteworthy candidates for facilitating both the transition from juvenile to adult phases transition and seasonal shift from vegetative to reproductive phase transition. This has been demonstrated in more and more woody plants (Fig. 1B).

### Aging pathway genes *miR156* and *SPLs* in woody plants

With the abundance of comparative genomic studies, *miR156* and *SPL* genes are increasingly identified in woody plants. This provides evidence to support that the miR156-*SPL* aging pathway is evolutionarily conserved, at least at gene sequence level, in woody plants (Wang, et al. 2011). For example, *miR156* exhibits high expression levels in young seedlings, which gradually decline with age during the transitions from the juvenile to the adult phase across various species, including Philippine acacia (*Acacia confusa*), Cole’s wattle (*Acacia colei*), blue gum (*Eucalyptus globulus*), ivy (*Hedera helix*), German oak (*Quercus acutissma*), Canadian poplar (*Populus* × *canadensis*), Chinese crabapple (*Malus hupehensis*), trifoliate orange (*Poncirus trifoliata*), kiwifruit (*Actinidia deliciosa*), Chinese plum (*Prunus mume*), and apple (*Malus domestica*) (Fang and Wang 2021; Feng, et al. 2023; Wang, et al. 2011). However, the complexity and heterozygous nature of woody plants have hindered comprehensive functional analysis of miRNAs and *SPL* genes, resulting in slow progresses in functional gene studies (Feng, et al. 2023; Jerome Jeyakumar, et al. 2020; Ma, et al. 2021; Zhao, et al. 2023). Of previous reports, the overexpression of *miR156* in Canadian poplar induced phenotypic changes, such as reduced internode length, plant height, and leaf size, and prolongs the juvenile phase by down-regulating target genes *SPL3* and *SPL9* (Wang, et al. 2011).

Theoretically, the miR156-SPL module in aging pathway plays a significant role in regulating the length of juvenility. This offers potentials to shorten juvenile period by primarily reducing the expression of the *miR156* RNA using interference (RNAi) or Clustered Regularly Interspaced Short Palindromic Repeats/CRISPR-associated protein 9 (CRISPR/Cas9). Alternatively, increasing expression of *SPL*s is another approach.

Surprisingly, neither approach has been successfully used for FasTrack Breeding in woody plants. Nevertheless, with the continuous development and application of new biotechnological tools, understanding of the functions and regulatory mechanisms of *miR156* and *SPL* genes in woody plants is expected to be enhanced. This may finally provide important insights and approaches for developing precocious flowering woody plants by manipulating the expression of targets in the aging pathway. Notably, the miR156-SPL module is not independent; many other factors interact with this module, including photosynthesis and hormone pathways (Pan, et al. 2023; Song, et al. 2024). This may cause a little uncertainty on a case-by-case basis when *miR156* or *SPL* is applied to shorten juvenile period. Regardless, to date, there has been no successful case of FasTrack Breeding by manipulating the expression of the miR156-SPL module in woody plants.

### *PEBP* genes in plant flowering phase transition

In angiosperms, the PEBP family consists of three phylogenetically distinct groups: FT-like proteins, TFL1-like [or CENTRORADIALIS (CEN)-like] proteins, and MOTHER OF FT AND TFL1 (MFT)-like proteins. Additionally, the BROTHER OF FT AND TFL1 (BFT)-like proteins represent a separate subclade within the TFL1 group (Karlgren, et al. 2011). Among these, FT and TFL1 play crucial, antagonistic roles in controlling the flowering transition and shaping plant architecture, largely through their expression balance (Moraes, et al. 2019). In contrast, MFT’s role in flowering is not broadly conserved; it is primarily recognized as a seed-preferential gene regulating germination (Cai, et al. 2023; Lu, et al. 2023; Nakamura, et al. 2011; Tao, et al. 2014; Xi and Yu 2010). Similarly, *BFT* appears to act redundantly with *TFL1*, and its function remains poorly characterized (Yoo, et al. 2004). Due to their predominant and well-established roles in flowering time control, this review will concentrate on the FT and TFL1 clades.

Annual and biennial plants, unlike many perennial plants, do not have a multiple-year juvenile period. In these plants, it has been extensively demonstrated that increased expression of *FT* or decreased expression of *TFL1* can promote flowering by shortening the vegetative growth period. Perennial plants undergo repeated cycles of vegetative growth, dormancy, and flowering, and many have a long juvenile period during which they cannot flower regardless of favorable environmental conditions. While numerous reports indicate that increased expression of *FT* or decreased expression of *TFL1* can promote flowering in adult-phase plants, there are not many in-depth to verify that increased expression of *FT* or decreased expression of *TFL1* can shorten the juvenile period in seedlings (Table 1).

*FT* genes integrate signals related to both plant age and environmental conditions and play multifaceted roles. They regulate the timing of first-time and seasonal flowering, manage dormancy cycles, and balance growth and reproductive efforts (André, et al. 2022; Hsu, et al. 2011; Pin and Nilsson 2012). In perennials, different *FT* homologues may function slightly differently. For instance, in *Populus* species, two *FT* genes, *FT1* and *FT2*, which share 91% amino acid sequence similarity, play distinct roles. The mRNA levels of *FT1* and *FT2* increase in leaves as the plant transitions from juvenile to reproductive stages, indicating their involvement in this developmental switch. During seasonally periodic flowering, *FT2* is central to the photoperiodic pathway, being expressed during the summer, while *FT1* is induced in buds during winter chilling. *FT2* facilitates vegetative growth in spring and summer and helps regulate the entry into dormancy, whereas *FT1* expression induces dormancy release (André, et al. 2022; Hsu, et al. 2011; Zhang, et al. 2010).

*TFL1* genes function oppositely to *FT* as floral repressors, likely delaying the transition from the juvenile to adult phase and from vegetative to reproductive development. *TFL1* and *FT* compete for FD protein (Zhu, et al. 2020). High expression of *TFL1* attributes to maintenance of juvenile phase and vegetative vigor at adult phase. This is typical of woody perennial plants. For example, in kiwifruit, two specific *TFL* genes, *AcTFL* and *AcTFL4*, are highly expressed in actively growing tissues, such as shoot tips and newly forming leaves (Voogd, et al. 2017). This suggests that these maintain the plant in its vegetative growth state, preventing premature transition to the reproductive stage.

Together, PEBP family genes are, to date, among the most effective genes for FasTrack breeding. Increasing the expression of *FT* or down-regulating the expression of *TFL1* can induce early flowering thus accelerating breeding cycles in woody plants. For instance, constitutive *FT* expression reduces the juvenile phase in transgenic seedlings of citrus, plum, and poplar (Callahan, et al. 2016; Endo, et al. 2020; Hoenicka, et al. 2014). Further investigation into other PEBP family members for modulating flowering time is needed and will be vital for developing climate-resilient crops.

### Additional flowering-promoting genes

In addition to the PEBP family, inflorescence identity genes, such as *LFY*, *AP1*, and *FRUITFULL* (*FUL*), play essential roles in flowering (Fig. 1B). Both *LFY* and *AP1* are critical flower meristem identity genes. Constitutive expression of *Arabidopsis LFY* or *AP1* genes yielded transgenic plants with fertile flowers and fruits within the first year, in contrast to the seedling derived *citrus* trees, which usually have a prolonged juvenile phase of 6–20 years (Peña, et al. 2001). Similarly, overexpression of *AP1 *via an anther culture system led to the early flowering of transgenic poplar individuals (Yang, et al. 2017). Transgenic grapefruit (*Citrus paradisi* Macf.) and sweet orange (*Citrus sinensis* [L.] Osbeck) plants expressing *CsAP1* or *CsLFY* under the *RESPONSE TO DESICCATION 29A* (*AtRD29A*) promoter exhibited early flowering under non-inductive conditions, producing viable seeds and underscoring the potential of these genes in reducing generation time (Orbovic, et al. 2021). Overexpression of *BpAP1* in birch induced early flowering within 2 months; this early flowering trait was inherited by subsequent generations, albeit accompanied by dwarfism (Huang, et al. 2014). Additionally, the silver birch (*Betula pendula*) MADS-box gene *BpMADS4*, which is phylogenetically related to *Arabidopsis FUL* and *AP1*, plays a pivotal role in maintaining inflorescence meristem identity and facilitating floral meristem specification in birch (Elo, et al. 2007, 2001). In apple (*Malus* × *domestica* Borkh.), constitutive overexpression of *BpMADS4* resulted in early flowering, with multiple transgenic lines consistently displaying flowers during in vitro propagation and continued flower development upon transfer to the greenhouse, suggesting the potential of *BpMADS4* for precocious flowering in apple (Flachowsky, et al. 2007a, b; Weigl, et al. 2015a, b). In European pear (*Pyrus communis*), *BpMADS4* overexpression triggered flowering within 6–18 months when grafted onto quince (*Cydonia oblonga*) rootstock (Tomes, et al. 2023). These studies collectively highlight the potential of *BpMADS4* in enhancing flowering in woody perennials. Occasionally, increasing *AP1* expression does not result in obvious early flowering, for example, overexpression of *BpMADS4* in poplar (*Populus tremula* L.) and overexpression of *JcAP1* in *Jatropha* did not induce early flowering (Hoenicka, et al. 2008; Tang, et al. 2016). These findings collectively suggest that *LFY* and *AP1* are candidate genes in regulating flowering time, albeit with variation of the effects of these genes across species and genotypes.

MADS-box genes play significant roles in the formation of floral meristems and organs (*e.g.*, male and female gametophyte), the control of floral transition and flowering time, and the development of seed and fruit (Becker and Theissen 2003; Causier, et al. 2002; Garcia-Maroto, et al. 2003; Gramzow and Theissen 2010, 2013; Heijmans, et al. 2012; Masiero, et al. 2011; Ng and Yanofsky 2001; Parenicova, et al. 2003; Theissen, et al. 2000). Among the proposed flowering-pathway gene networks (Fornara, et al. 2010; Hill and Li 2016), *SOC1* is, alongside *AP1*, another MADS-box gene that has significant impacts on plant reproductive development and flowering time control through the conserved *CO-FT-SOC1* module (Liu, et al. 2013). As a downstream gene of *FT* and an upstream gene of *LFY* and *AP1*, *SOC1* has been demonstrated to be an effective integrator and promoter of flowering (Andres and Coupland 2012; Fornara, et al. 2010; Lee and Lee 2010). 

The role of *SOC1* in promoting flowering has been documented in various plant species, including *Arabidopsis* (Immink, et al. 2013; Lee, et al. 2000), rice (*Oryza sativa*) (Lee, et al. 2004), grapevine (*Vitis vinifera* L.) (Sreekantan and Thomas 2006), wheat (*Triticum aestivum* L.) (Shitsukawa, et al. 2007), poplar (Bruegmann and Fladung 2019; Gómez-Soto, et al. 2021), sweet orange (Tan and Swain 2007), soybean (*Glycine max*)(Han, et al. 2021; Zhong, et al. 2012), orchid (*Dendrobium Chao* Praya Smile) (Ding, et al. 2013), kiwifruit (Voogd, et al. 2015), maize (*Zea mays*) (Alter, et al. 2016; Song, et al. 2021), cotton (*Gossypium arboreum* L.) (Zhang, et al. 2016), tree peony (*Paeonia* × *suffruticos*) (Wang, et al. 2015), alfalfa (*Medicago sativa* L.) (Jaudal, et al. 2018), and tomato (*Solanum lycopersicum*) (Zahn, et al. 2023). Additionally, *SOC1* is also involved in floral development, controlling inflorescence-branching development, and managing chilling requirements for dormancy break (Gómez-Soto, et al. 2021; Ruokolainen, et al. 2011; Song, et al. 2023; Trainin, et al. 2013; Zahn, et al. 2023).

To meet the challenges of climate change, the MADS-box gene family offers promising genes for FasTrack breeding by shortening juvenile phases (Table 1). Constitutive expression of the *FUL*-like *BpMADS4* gene from silver birch enabled transgenic apple seedlings to flower within 1 year, by passing the typical 4–5 year juvenile period (Flachowsky, et al. 2011). A similar reduction in juvenility was achieved in citrus seedlings through the constitutive expression of *AP1* and *LFY* (Peña, et al. 2001). The role of other flowering-promoting genes in this context remains an important area for future research.

## Generation of FasTrack breeding materials

**Fig. 2:**
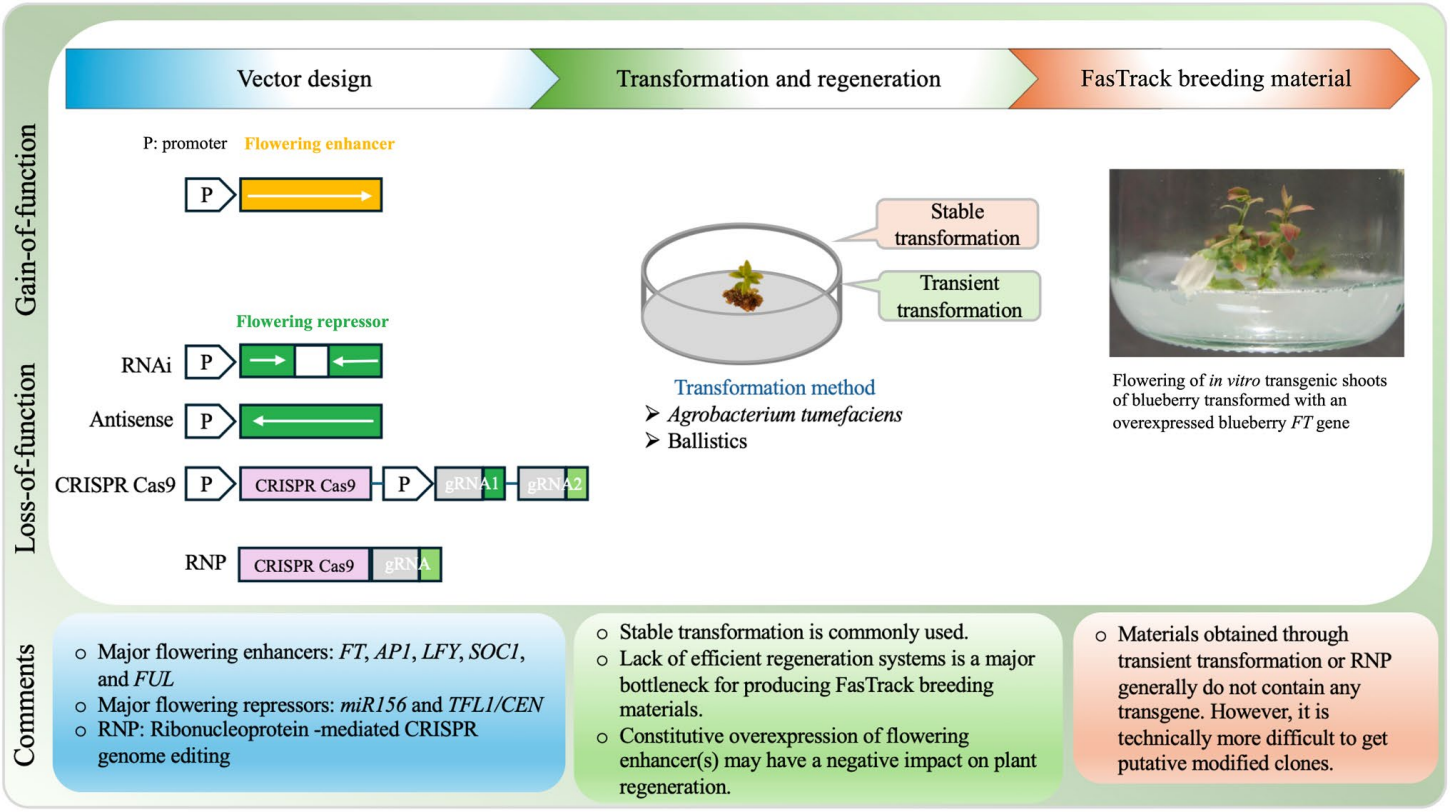
Approaches to produce early flowering materials for FasTrack breeding

Two common strategies to develop FasTrack breeding materials involve gain-of-function by increasing the expression of floral initiators (*e.g.*, *FT*, *AP1*, *LFY*, and *FUL*) and loss-of-function by repressing the expression of floral repressors (*e.g.*, *miR156* and *TFL1*) (Fig. 2). These strategies can be implemented through either stable or transient (*e.g.*, virus-mediated gene expression or silencing systems) transformation methods (Table 1). To date, stable transformation systems have been more widely used and are generally preferred over transient expression methods due to their greater effectiveness, stability, and reliability. Additionally, floral enhancers have been more extensively studied than floral repressors (Table 1).

### Loss-of-function strategy for floral repressors

Gene knock-down using RNA interference (RNAi) and gene knock-out using CRISPR/Cas9-mediated gene editing are major approaches for loss-of-function strategies. *TFL1* and *miR156* are two key floral repressors that can be targeted to produce FasTrack breeding materials using these strategies. With the advancement of the CRISPR/Cas9 technologies, these two candidate genes have become particularly desirable for producing FasTrack breeding materials due to the high potential for generating edited plants without transgene integration. Although there have been successful reports of using RNAi and CRISPR/Cas9 to target these two genes, further evidence is needed to demonstrate that reducing the expression of *TFL1* or *miR156* can shorten the juvenile period in modified seedlings (Table 1).

### Gain-of-function strategy for floral enhancers

A constitutive gene expression system is commonly used in gain-of-function to investigate floral activator genes. Using this system, the functions of many floral enhancers, such as *FT*, *LFY*, *FUL*, and *AP1* from various plant species, have been evaluated (Table 1). While constitutive expression, particularly overexpression driven by the CaMV 35S promoter, often induces early flowering, it can also lead to abnormal phenotypic changes, including dwarfing and stunted growth (Endo, et al. 2005; Shen, et al. 2012; Tränkner, et al. 2010). Moreover, it is worth noting that overexpression of floral activators can reduce regeneration frequency or inhibit the generation of transgenic lines with actively expressed target genes. For instance, transcriptomic analysis has revealed numerous differentially expressed genes related to phytohormone synthesis in *FT*-overexpressing plants (Walworth, et al. 2016). It is well known that hormones play significant roles in plant transformation and regeneration.

Non-constitutive promoters such as tissue-specific or inducible promoters provide alternative approaches to alleviate the negative impact of the constitutive promoters. Of these promoters, the *Arabidopsis AtSUC2* (*SUCROSE TRANSPORTER* 2) promoter, known for its phloem-specific activity, has successfully been used to express an *FT* gene for tissue-specific expression in Carrizo citrange rootstocks, leading to flowering within 16 months without reducing plant vigor (Soares, et al. 2020). Interestingly, a study with transgenic apple trees using the *MdFT1* gene under the *AtSUC2* promoter did not result in early flowering, although the transgenic plants exhibited robust growth (Tränkner, et al. 2010). These results suggest that it is possible to use tissue-specific promoters to promoter flowering without significant penalty on overall plant growth, although the effectiveness of these promoters may vary across plant species and genotypes. Similarly, several studies have demonstrated that inducible promoters, *e.g.,* heat shock protein promoters, are capable of driving early flowering under inducible conditions (Table 1) (Azeez and Busov 2019; Hoenicka, et al. 2016; Zhang, et al. 2010).

## FasTrack breeding approaches

**Fig. 3:**
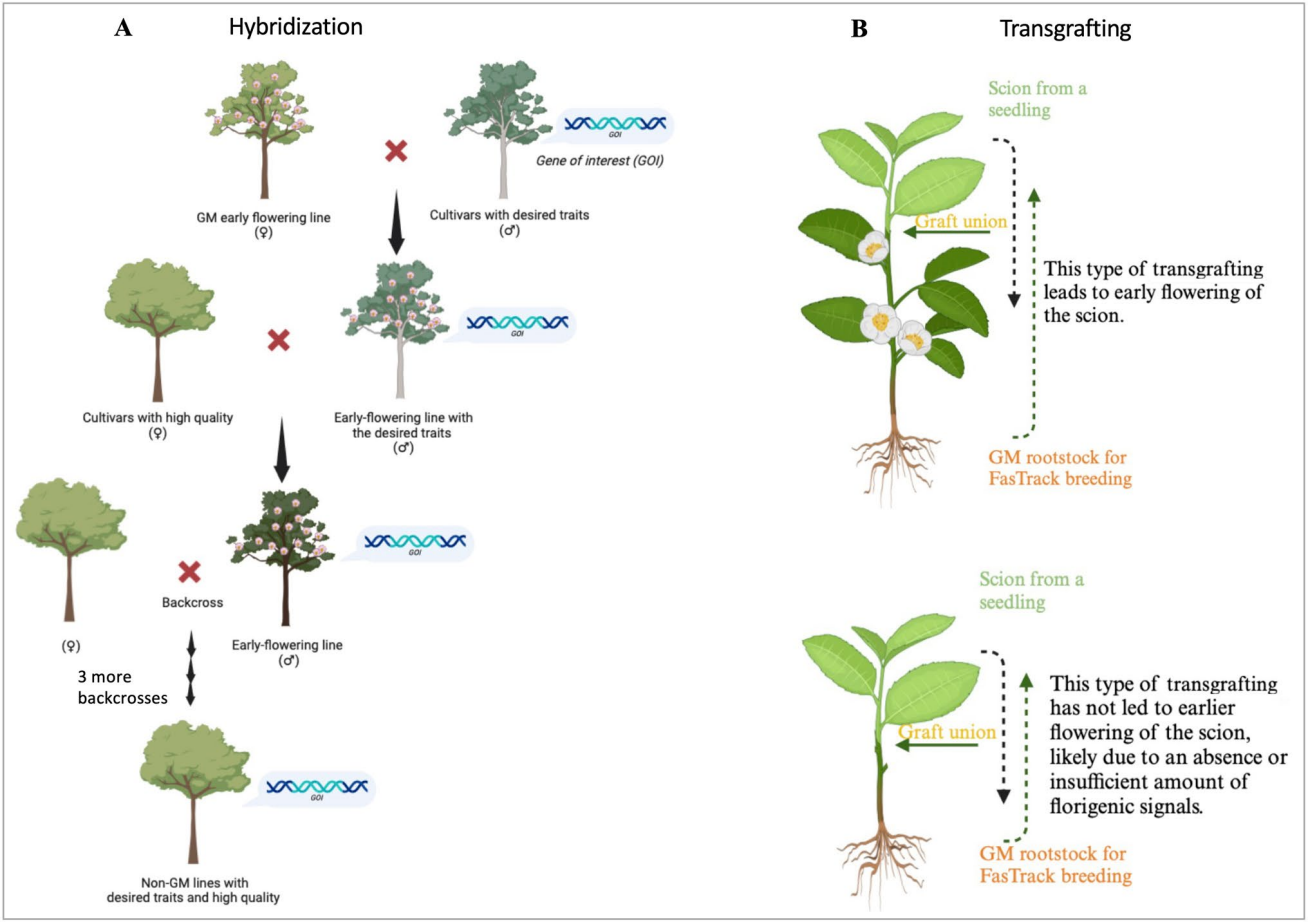
FasTrack breeding in woody plants using hybridization or transgrafting. **A** Schematic of the hybridization approach. **B** Schematic of the transgrafting approach. Panel A was created with BioRender

FasTrack breeding materials, ideally elite varieties or genotypes, are genetically engineered to include specific genes, such as floral activators and repressors, to shorten the juvenile period in plants. There are two basic methods for utilizing these FasTrack materials to accelerate the breeding process: hybridization and transgrafting (Fig. 3). Hybridization is a fundamental plant breeding technique that facilitates the exchange of multiple genes between parent plants, including the engineered gene targets. The progenies resulting from this process are expected to inherit the engineered genes and, as a result, exhibit a shortened juvenile period. Transgrafting, on the other hand, refers to a specialized form of grafting that involves the use of either a transgenic scion or a transgenic rootstock.

### Hybridization approach

In the hybridization approach, FasTrack breeding materials are crossed with a parent possessing the desired gene(s) or trait(s). After the initial cross, progeny seedlings exhibiting early flowering and the targeted desirable trait(s) or gene(s) are selected for further crossing and backcrossing with a targeted elite cultivar to incorporate these traits (Fig. 3) (Callahan, et al. 2016). To date, this approach has been demonstrated in plum, apple, and citrus.

In plum, the introduction of the *PtFT* gene resulted in early flowering lines, while an RNAi vector conferred resistance to the plum pox virus (PPV) in transgenic lines. These transgenic lines, derived from seedling explants, are generally considered less commercially viable due to their overall trait profiles compared to elite cultivars. However, using the FasTrack breeding approach (Fig. 3), crossing and backcrossing, assisted by *PtFT*-induced early flowering and the presence of the PPV-silencing transgene, accelerated the integration of PPV resistance into commercial cultivars, where stable transformation systems are otherwise lacking (Callahan, et al. 2015; Scorza, et al. 2013; Srinivasan, et al. 2012).

In apple, a transgenic line of ‘Pinova’ overexpressing the *BpMADS4* gene enabled fast-track breeding. This transgenic line was crossed with wild species to introduce resistance genes against diseases, such as fire blight, apple scab, and powdery mildew, using marker-assisted selection. The resulting progenies, which possess both the *BpMADS4* gene for early flowering and specific disease resistance genes, provide valuable material for efficiently introgressing disease resistance traits into various apple cultivars (Flachowsky, et al. 2011; Flachowsky, et al. 2007a, b; Hanke, et al. 2012; Le Roux, et al. 2012; Patocchi, et al. 2021; Schlathölter, et al. 2018; Weigl, et al. 2015a, b).

In citrus, to accelerate the breeding process for condensed tristeza virus resistance, early flowering citrus lines overexpressing *CiFT*, assisted by marker-assisted breeding, were used to incorporate the resistance gene from trifoliate orange into commercial citrus cultivars (Endo, et al. 2020).

### Grafting and transgrafting approach

Grafting, including transgrafting, is a traditional practice commonly used in horticulture, especially for woody plants (Goldschmidt 2014; Habibi, et al. 2022; Song, et al. 2015). The interactions between a scion and a rootstock, through the exchange of various signals and compounds, can significantly impact the grafted plant's traits, such as abiotic and biotic tolerance, yield, and fruit quality (Calderón, et al. 2021; Goldschmidt 2014; Habibi, et al. 2022; Nanda and Melnyk 2018). Transgrafting specifically refers to grafting that involves the use of transgenic materials, either in the scion or the rootstock (Song, et al. 2015).

Rootstocks can influence both vegetative and reproductive growth of scions (Webster 1995). For example, in apple trees, grafting is commonly employed to regulate plant size, flowering time, flower bud formation, yield, and fruit quality (Fazio, et al. 2014; Habibi, et al. 2022; Seleznyova, et al. 2008; Souza, et al. 2018; van Hooijdonk, et al. 2011; Webster 1995). Grafting is often employed to shorten the juvenile period of apple trees (Fischer 1994). The Malling 9 (M.9) dwarfing rootstock, for instance, not only induces dwarfism but also reduces the juvenile period and enhances flowering in the scion. Research suggests that dwarfing signals may originate from the vascular system. RNA sequencing studies have shown higher expression levels of genes, such as *MdFT1/2*, *MdBFTa/b*, *MdCO*, *MdGI*, and *MdSOC1* in dwarfing rootstocks (M.9 and Malling 27) compared to the standard rootstock M.793, which may have contributed to earlier flowering (Foster, et al. 2014). Although many mobile substances, including mineral nutrients, sucrose, proteins, miRNAs, RNAs, and hormones, can act as long-distance signals in grafted plants (Habibi, et al. 2022), it remains uncertain which of these are the key factors determining flowering. The leading candidates are florigen and hormones (Cho, et al. 2022; Huang, et al. 2018a, b; Kawaguchi, et al. 2024; King and Ben-Tal 2001; King and Evans 2003; Shalit, et al. 2009).

*FT* is currently considered the leading candidate for the florigen, while *TFL1* is the primary candidate for the antiflorigen due to its opposing roles (Huang, et al. 2018a, b; Matsoukas, et al. 2012; Putterill and Varkonyi-Gasic 2016; Shalit, et al. 2009; Turck, et al. 2008). This lays the foundation for the FasTrack breeding approach, which involves using transgenic rootstock with enhanced *FT* expression (referred to as enFT-rootstock) or repressed *TFL1* expression (referred to as reTFL1-rootstock) to shorten the juvenile period of scions. In transgrafting experiments using FT-rootstocks, increased flowering has been observed in nontransgenic scions of blueberry and Jatropha (Song, et al. 2019; Ye, et al. 2014). However, this effect was not observed in apple, cassava, and poplar enFT-rootstocks, where the flowering of nontransgenic scions was not accelerated (Odipio, et al. 2020; Wenzel, et al. 2013; Zhang, et al. 2010).

The varying outcomes of transgrafting using enFT-rootstocks are likely due to differences in the strength of the FT source within the rootstocks. In successful cases, a significant number of leaves were retained on the enFT-rootstocks, whereas, in unsuccessful cases, leaves were either removed or kept to a minimum. Maintaining some leaves on enFT-rootstocks appears to be crucial for inducing flowering in nontransgenic scions of blueberry enFT-rootstocks (Song, et al. 2019). However, this point remains debatable, as evidenced by the Malling 9 (M.9) dwarfing rootstock, which promoted flowering without retaining leaves on the rootstock itself (Foster, et al. 2014). Questions remain regarding the nature of the long-distance signals driven by *FT* overexpression (Huang, et al. 2018a, b; Notaguchi, et al. 2008). It is surprising that constitutive expression of *VcFT* did not lead to a significant increase in *VcFT* expression in transgenic blueberry roots, despite the presence of numerous differentially expressed genes (Song, et al. 2019).

Research on the long-distance mobility of florigen members in grafted plants, apart from *FT*, is limited (Huang, et al. 2018a, b; Notaguchi, et al. 2008). In apple, rootstocks with suppressed expression of the apple *TFL1* gene (*MdTFL1-1*) through RNAi showed precocious flowering (Flachowsky, et al. 2012). However, transgrafting non-transformed seedlings from traditional crosses onto these early flowering rootstocks did not result in early flowering of the scions. This suggests that either *MdTFL1-1* is not systemically transported, as proposed by the authors, or the transported *MdTFL1-1* small interfering RNAs (siRNAs) are insufficient to cause a noticeable phenotypic change (Flachowsky, et al. 2012). In fact, the long-distance transfer of transgene-induced siRNAs has been demonstrated in transgrafted plants (Alburquerque, et al. 2023; Zhao and Song 2014).

In grafted plants, phloem-mobile *miR156* play a role in the systemic regulation of interactions between the scion and rootstock as well as within the scion itself (Zhao, et al. 2020). Among the flowering-pathway genes, *miR156* is known to be crucial in maintaining the juvenile phase of plants. It is also graft-transmissible, as demonstrated in potato heterografts, where it was shown to act as a graft-transmissible signal that influences plant architecture and tuberization (Bhogale, et al. 2014). To date, investigations into using transgenic rootstocks down-regulating *miR156* to regulate the juvenile period or flowering time of nontransgenic scions in woody plants are still lacking.

In summary, despite the ongoing debate about which molecules function as long-distance transmissible florigen and antiflorigen, it is possible to shorten the juvenile period through grafting and transgrafting.

### FasTrack breeding for gene editing

An ideal gene-editing system achieves high on-target activity with minimal off-target effects and operates without integrating foreign DNA (transgenes). Two primary strategies can generate these transgene-free plants. The first involves segregating out CRISPR/Cas9 transgenes through genetic crosses in subsequent generations. While effective for seed-propagated crops, this method is less suitable for clonal species unless a FasTrack system is available to rapidly excise transgenes (Song 2025; Wenzel, et al. 2013).

The second strategy employs transient delivery methods, such as viral vectors or pre-assembled ribonucleoproteins (RNPs), to directly produce transgene-free edited plants (Andersson, et al. 2018; Liang, et al. 2016; Ma, et al. 2020; Metje-Sprink, et al. 2018). These methods introduce editing components into regenerable cells, often resulting in chimeric plants, but are applicable to both seed and clonal crops. A major limitation, however, is the low efficiency of plant regeneration, particularly from challenging systems like protoplasts.

**Fig. 4:**
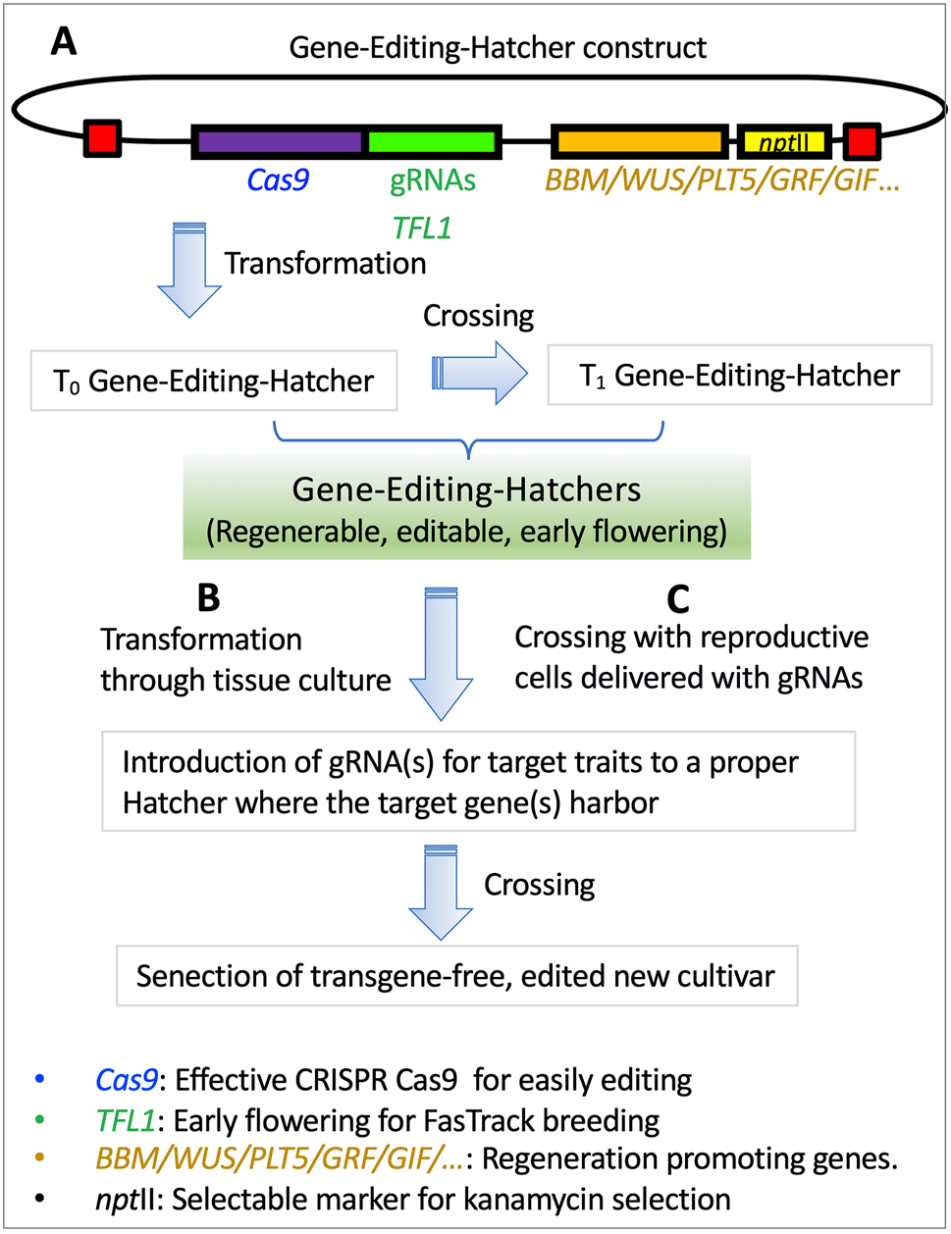
Schematic of the “Gene Editing Hatchers” strategy for accelerated breeding and editing. **A** Construct delivery and stock expansion: initial transformation generates the founder “Gene Editing Hatcher” plants. These plants are characterized by early flowering, which facilitates rapid cross-pollination with diverse genotypes. This allows for the efficient introgression of the editing constructs, enabling quick expansion of a reusable “Hatcher” stock population. **B** Editing via tissue culture: “Hatcher” lines provide an amenable platform for gene editing. They can be subjected to tissue culture for a second transformation with sequence-specific gRNAs. Following editing, the original transgenes (*e.g.*, for early flowering and the editing machinery) are segregated out through genetic crossing, producing edited plants with no foreign DNA. **C** Direct *in planta* editing: The “Hatcher” system enables a tissue culture-free editing pipeline. gRNAs are delivered directly into the reproductive tissues (*e.g.,* gametes, pollen grains, and meristems) of “Hatcher” plants, generating edited progeny without the need for a tissue culture phase. *BBM*: *BABY BOOM*; *WUS*: *WUSCHEL*; *PLT5*: *PLETHORA 5*; *GRF*: *GROWTH-REGULATING FACTOR*; *GIF*: *GROWTH-REGULATING FACTOR-INTERACTING FACTOR*

To overcome the key bottlenecks in woody fruit crops, inefficient regeneration, and the mandatory removal of transgenes, we propose a novel “gene-editing hatcher” system. As shown in Fig. 4, each hatcher integrates two components: 1) a reusable CRISPR/Cas9 system that shortens the juvenile phase by knocking out *TFL1* (FasTrack breeding) and enables additional edits, and 2) a regeneration-promoting gene cassette to drastically improve transformation efficiency. Crucially, the FasTrack system is designed to facilitate the subsequent removal of all transgenic cassettes, yielding non-chimeric, transgene-free edited lines ready for integration into elite cultivars (Fig. 4B, C). It should be noted that this “gene-editing hatcher” system described here, currently under development for grapevine (our unpublished data), remains a conceptual proposal rather than any established method.

## Conclusions and future prospect

For developing climate-resilient plants, FasTrack breeding is particularly advantageous for species with long juvenile periods, as it enhances breeding efficiency by shortening each generational cycle. Proof-of-concept studies in various plant species have validated this approach. Research into flowering pathways has identified key candidate genes for engineering FasTrack lines, including floral activators (*e.g*., *FT*, *LFY*, *AP1*, *FUL*, and *SOC1*) and repressors (*e.g*., *miR156* and *TFL1*). Among these, *FT* and *TFL1* are especially prominent. A primary technical challenge in producing such materials is the difficulty of regenerating transgenic plants, as the introduction of flowering-pathway genes can directly interfere with the regeneration process. Successfully engineered FasTrack breeding lines can subsequently be utilized either as parental lines or as rootstocks to accelerate conventional breeding programs.

This review has covered key literature on flowering pathways in woody plants, though the breadth of the field means some studies may have been unintentionally omitted. It is also important to note that non-flowering pathways, including those regulated by hormones and sugars, hold considerable promise for shortening the juvenile period (Song 2025). Looking forward, the integration of genomics, marker-assisted selection, and gene-editing technologies is poised to identify and deploy novel strategies for significantly accelerating breeding programs.
